# Maternal Antibody Transmission in Relation to Mother Fluctuating Asymmetry in a Long-Lived Colonial Seabird: The Yellow-Legged Gull *Larus michahellis*

**DOI:** 10.1371/journal.pone.0034966

**Published:** 2012-05-09

**Authors:** Abdessalem Hammouda, Slaheddine Selmi, Jessica Pearce-Duvet, Mohamed Ali Chokri, Audrey Arnal, Michel Gauthier-Clerc, Thierry Boulinier

**Affiliations:** 1 Département des Sciences de la Vie, Faculté des Sciences de Gabès, UR, Biodiversité & Valorisation des Bioressources en Zones Arides, UR11ES86, Gabès, Tunisia; 2 Centre d’Ecologie Fonctionnelle et Evolutive, CNRS-UMR 5175, Montpellier, France; 3 Centre de Recherche de la Tour du Valat, Le Sambuc, Arles, France; Institute of Marine Research, Norway

## Abstract

Female birds transfer antibodies to their offspring via the egg yolk, thus possibly providing passive immunity against infectious diseases to which hatchlings may be exposed, thereby affecting their fitness. It is nonetheless unclear whether the amount of maternal antibodies transmitted into egg yolks varies with female quality and egg laying order. In this paper, we investigated the transfer of maternal antibodies against type A influenza viruses (anti-AIV antibodies) by a long-lived colonial seabird, the yellow-legged gull (*Larus michahellis*), in relation to fluctuating asymmetry in females, i.e. the random deviation from perfect symmetry in bilaterally symmetric morphological and anatomical traits. In particular, we tested whether females with greater asymmetry transmitted fewer antibodies to their eggs, and whether within-clutch variation in yolk antibodies varied according to the maternal level of fluctuating asymmetry. We found that asymmetric females were in worse physical condition, produced fewer antibodies, and transmitted lower amounts of antibodies to their eggs. We also found that, within a given clutch, yolk antibody level decreased with egg laying order, but this laying order effect was more pronounced in clutches laid by the more asymmetric females. Overall, our results support the hypothesis that maternal quality interacts with egg laying order in determining the amount of maternal antibodies transmitted to the yolks. They also highlight the usefulness of fluctuating asymmetry as a sensitive indicator of female quality and immunocompetence in birds.

## Introduction

Most organisms face a dangerous world, in which parasitic species outnumber host species [1], and vertebrates have evolutionarily responded to this threat by developing a complex immune system in which antibodies provide tailored protection against the particular pathogens encountered. In vertebrates, antibodies are known to be maternally transferred, thus conferring passive immune protection against parasites faced by the offspring after birth or hatching and potentially impacting offspring growth and survival [2]–[4]. In birds, the capacity to synthesize and transmit antibodies to offspring *via* egg yolks, which could be related to female quality, may vary among females according to a multitude of factors [2], [5]–[7]. Some of these may be proximate factors, such as current female condition and nutritional status, but others may be historical factors that acted during early life stages. Female quality could thus affect maternal antibody transfer in two ways: through the overall capacity to produce and deposit antibodies and their distribution within the clutch. Overall, higher quality females should be more capable of synthesizing immunoglobulins and depositing them in egg yolks than lower quality ones [2], [7], [8]. At the level of the clutch, patterns become more complicated and have received far less attention. The quantity of maternal antibodies deposited is known to vary among eggs according to egg laying order, but the directionality of this relationship depends on the reproductive strategy adopted [9]–[11].

In altricial birds, two opposite reproductive strategies evolved in response to asynchronous egg hatching in circumstances of unpredictable food availability [12]. In many species, females may seek to improve whole brood survival by increasing the allocation of maternal resources, such as hormones and immunoglobulins, to the last-laid egg, thus reducing the effect of hatching asynchrony on nestling competition and improving the survival probability of the youngest hatchling [13], [14]. However, species more commonly demonstrate adaptive brood reduction. In this system, females may improve the survival probability of the first nestlings by allocating more resources to the first-laid eggs, which are likely to have the highest reproductive value, and thus sacrifice the ones that hatch last [15], [16]. In both systems, the ability of females to maintain a differential antibody transmission to eggs according to their laying order, and thus the extent of the laying order effect, would reflect their quality. In the case of a brood reduction strategy, two opposite trends could be predicted. First, lower quality females may be less able to control antibody deposition into egg yolks in accordance with laying order, resulting in a reduced laying order effect. Alternatively, because lower quality females could have fewer antibodies to deposit in their eggs, they may transmit most of this amount to the first eggs, at the cost of the last ones. This would result in a more pronounced laying order effect.

Female quality is often estimated by determining the level of fluctuating asymmetry (FA), i.e. the random deviation from perfect symmetry in bilaterally symmetric morphological traits [17], [18]. FA reflects deficiency in the early-life developmental processes, i.e. developmental instability, due to stressful conditions such as food limitation, parasitism and other challenges [19]–[23]. It is generally negatively correlated with fitness-related traits [23] and is increasingly viewed as a reliable morphological indicator of individual quality [24]–[26]. In general, more asymmetric birds have lower survival and breeding success than symmetric ones. Using this line of reasoning, FA could be used as an indicator of female immunocompetence and one could expect females with greater asymmetry to produce fewer antibodies and to transmit lower amounts of antibodies to their eggs than do the more symmetric ones.

We investigated the extent to which maternal quality, as estimated through fluctuating asymmetry, contributes to within-clutch variation in yolk antibodies using avian influenza in the yellow-legged gull (*Larus michahellis*) as a model. The yellow-legged gull is a suitable model for such an investigation as it is a long-lived species, demonstrates asynchronous egg hatching [27], [28], and appears to adopt the brood reduction strategy [29].

## Methods

### Ethics Statement

This study complies with the current Tunisian laws regarding ethics and animal use for scientific purposes. It was approved by the “Forest Department” in the Tunisian Ministry of Agriculture, which is the relevant authority in charge of wildlife use and conservation in Tunisia, through the permit number 518-28/02/2009.

In the Mediterranean region, the yellow-legged gull is an abundant species, and is subject to population control measures [30]–[32], which makes it amenable to adult capture and egg sampling. Upon capture, birds were kept in a safe position as is standard practice in bird ringing studies. Blood samples were taken from the brachial vein using a sterile syringe and the puncturing site was accurately disinfected. All individuals were released as soon as possible. After being released, the sampled birds behaved normally and resumed their normal breeding activities.

### Study Species and Area

The yellow-legged gull is a socially monogamous and semicolonial bird of the family *Laridae*. It is long-lived and shows strong interannual breeding site fidelity [33]. It typically lays one clutch per season, with a modal clutch size of 3 eggs, although replacement clutches are possible. Eggs are laid at 1–3 day intervals and hatching is typically asynchronous. Egg volume decreases as laying progresses, with markedly smaller last-laid eggs [27], [28]. Chicks are semiprecocial and remain around the nest for the first few days of life, after which they become highly mobile. In Tunisia, the yellow-legged gull is a common and abundant resident bird that nests on the numerous small islands along the coast, as well as in some coastal wetlands [34]. Our work was conducted during spring 2009 in two breeding colonies situated in the gulf of Gabès, south-eastern Tunisia: Sfax salina (34°42′28′′N−10°45′02′′E) and the small islets between Djerba island and the Zarzis peninsula (33°39′10′′N–10°58′59′′E). The yellow-legged gull in this area is often seen feeding on open air rubbish dumps and discards of commercial fisheries.

### Data Collection

#### Egg and blood sampling and morphological measurements

Data were collected between March 25^th^ and April 11^th^, 2009. Randomly selected nests were marked with small wooden stakes and checked every 1–2 days for egg collection. Each egg was marked according to its rank and laying date and then replaced by a dummy gull egg to prevent females from abandoning their nests. The eggs were brought to the laboratory on the day of collection for processing. Collected eggs were thus 1 to 2 days old and their yolk composition was not yet affected by embryonic development. The yolk of each collected egg was separated from the albumen, homogenized, and frozen at −20°C. Only the results from nests containing three eggs were considered in this study.

Following clutch completion, we trapped as many of the incubating parents as possible by means of noose-carpet traps placed on the nests. Each captured bird was marked with a patch of paint on the head to avoid resampling. Upon capture, birds were weighed (±20 g) with a spring scale (PESOLA®, Switzerland). Four bilateral morphological traits were then measured with a digital caliper (±.01 mm) on both the left (l) and right (r) sides: (1) Nalospi length (N), defined as the distance from the tip of the bill to the nostril, (2) lower mandible length (B), defined as the distance from the tip of the lower mandible to the corner of the mouth, (3) tarsus length (T), defined as the tarso-metatarsus length, and (4) middle toe length (P), defined as the distance from the first scale to the base of the nail of the middle toe. Measurements were always conducted by the same observer (A. Hammouda). In a number of birds, measurements were repeated three times to assess repeatability. The observer measured the target traits on one side and then on the opposite side, always following the same order: Nalospi length, lower mandible length, tarsus length, and middle toe length. This procedure was then repeated three times without knowledge of previous recorded values. All measurements were highly repeatable (*r*>0.99, *P*<0.0001 for all morphological traits). Before releasing the bird, a 1-ml blood sample was taken from the brachial vein using a sterile syringe. The blood sample was immediately transferred to a heparinized tube and maintained in a cooler at 4°C while in the field. Once in the laboratory, a subsample of blood (50–70 µl) was placed in microcapillary tubes, and centrifuged at 11 500 r.p.m. for 10 min. The haematocrit value was defined as the proportion of the microcapillaries that were occupied by red blood cells. Haematocrit value was regarded as an index of the health status of the bird [35], [36]. Whole blood was centrifuged at 2500 rpm for 15 min. The plasma and blood cells were frozen separately at −20°C until immunological analyses could be performed.

#### Sex determination

The sampled birds were sexed following a molecular method [37]. DNA was extracted from the blood using a DNeasy Blood & Tissue kit (Qiagen Inc., Valencia, CA) and used in PCR with primers 2550 Forward and 2718 Reverse to amplify introns from the CHD-*Z* and CHD-*W* genes, located on the avian sex chromosomes [38]. PCR fragments were then separated on an electrophoresis agarose gel. In this method, a single band of DNA on the gel indicated that a bird was a male, while two bands were present for females.

#### Immunological analyses

Anti-AIV antibodies in plasma and yolk samples were measured using a commercial competitive enzyme-linked immunosorbent assay (ELISA) developed for use in birds (ID Screen® Antibody Influenza A Competition, ID VET, Montpellier, France). The assay is designed to detect antibodies directed against the internal AIV nucleocapsid and thus it will detect all AIV subtypes. Plasma samples were used directly in the immunological assays. However, yolk antibodies were first extracted [39], [40]. Egg yolks were thawed and homogenized. A subsample of 800 mg of yolk was then diluted 1∶1 in phosphate-buffered saline solution (PBS) to which a few glass beads were added. The solution was shaken in a mill until a homogenous emulsion was obtained and an equal volume of reagent-grade chloroform was added to the mixture. The yolk-chloroform blend was then centrifuged at 16 000 rpm for 15 min and the clear supernatant was used in the immunological assays.

Plasma and yolk supernatant samples were diluted 1∶100 and incubated at 37°C for one hour. After a washing step, a peroxidase-marked conjugate was added to each well and the samples were incubated for 30 min at 21°C. The plates were then washed again, a substrate solution was added to each well, and the samples were incubated for 10 min at 21°C in the dark. Finally, a stop solution was added to each well in order to stop the reaction. Optical density (OD) was read at 450 nm using a spectrophotometer. A subset of samples were repeated, both within and across plates and we found that OD measurements were highly repeatable (within: *r* = 0.913, *F*_29,30_ = 22.84, *P*<0.0001; across: *r* = 0.85, *F*_33,24_ = 9.56, *P*<0.0001).

According to the kit instructions, the results were expressed as the percentage competition (PC) between the OD of the sample being tested and the mean OD of a negative control sample (NC), such that PC = (OD_specimen/_OD_NC_)×100. The percentage competition values were then transformed into percentage inhibition values (PI) using the formula: PI = 100−PC. The PI was used as measure of anti-AIV antibody concentration.

### Data Analyses

#### Fluctuating asymmetry and body condition

For each measured bilateral trait (B, N, T and P), we calculated the signed difference between the left and right sides (*l–r*), the absolute difference between left and right (|*l–r|*), and the average size [(*l+r*)/2]. To evaluate the possibility of anti-symmetry (i.e. a tendency away from bilateral symmetry), we checked for departures from normality of the distribution of the signed differences (*l–r*) using the Shapiro-Wilk test. To test for any directional asymmetry (i.e. biased to one side), a Student’s *t*-test was used to determine whether the mean of signed differences between left and right sides (*l–r*) was significantly different from zero [41]. If no directional asymmetry is present and the distribution of signed differences is normal, then the variation in these differences represents classical fluctuating asymmetry [41]. For all the four traits (B, N, T and P), we calculated a size-corrected index of fluctuating asymmetry: *FA_i_ = [(|l–r|)/((l+r)/2)]*. We then calculated a composite fluctuating asymmetry index FA for each female, by summing the FA_i_ values across the four traits: *FA = FA_B_+FA_N_+FA_T_+FA_P_*.

In order to obtain one composite measure of female body condition, a Principal Components Analysis was carried out on the following parameters: hematocrit value (%), clutch volume (cm^3^), and the residuals of the regression of body mass (g) on tarsus length (cm) as a measure of size-corrected female mass. Only factors whose eigenvalues exceeded 1 were retained (see Results). Egg volume was calculated using the following formula: egg volume (cm^3^) = 0.000476×length (mm)×width^2^ (mm) [42]. Larger eggs and clutches are supposed to be laid by females in better body condition [43].

#### Body condition, fluctuating asymmetry and patterns of maternal antibody transfer

The relationship between female body condition index (BCI), as a response variable, and fluctuating asymmetry score (FA), as an explanatory variable, was assessed by means of simple linear regression. We also used separate linear regressions to investigate the relevance of female BCI and FA as possible predictors of female anti-AIV antibody levels. In the latter regressions, female anti-AIV antibody level was arcsin-transformed to ensure the normal distribution of model residuals. Furthermore, in order to check if females with higher antibody levels transmitted higher amounts of antibodies to their eggs, we calculated the average antibody level (average PI) for each clutch and then regressed these average egg antibody levels (arcsin-transformed) on the plasma antibody levels of the corresponding mothers (arcsin-transformed). We also regressed the average egg antibody levels on the fluctuating asymmetry scores of the corresponding mothers to verify whether more symmetric females transmitted more antibodies to their eggs. As we were also interested in testing whether yolk antibody level varied within clutches according to egg laying order, we conducted a repeated-measures ANOVA on yolk antibody level as a function of nest identity and egg laying order (categorical variable with three classes: eggs 1, 2 and 3). A Duncan post-hoc test was conducted to identify significant differences. Moreover, in order to determine if the extent of intra-clutch variation in yolk antibody level varied according to the level of fluctuating asymmetry in the corresponding mothers, we calculated the coefficient of variation of yolk antibody levels (intra-clutch CV) for each clutch, and then tested for the significance of female fluctuating asymmetry score as a predictor of intra-clutch CV (log-transformed) by means of simple linear regression. Finally, we calculated the difference in antibody level between the first-laid and the last-laid egg in each clutch, and we checked whether this difference (log(x+10)-transformed) was related to maternal FA by means of linear regression. All statistical analyses and tests were carried out using SAS software [44]. All means are reported in the text ±1SE.

## Results

We were able to obtain complete data (blood and yolk antibody levels, morphological measurements, hematocrit values, and clutch volume) for a total of 18 anti-AIV antibody positive females. All females sampled were among the earliest-nesting females (day 1 to 8) in the study colony (egg laying occurred between days 1 and 8, with 1 is the first recorded laying date) and could thus be considered to be among the oldest as well. Anti-AIV antibody level (%) varied between 75 and 97 (mean = 90.96±1.21) in plasma samples and between 34 and 100 (mean = 80.58±2.03) in egg yolk samples. Female hematocrit (%) ranged from 39 to 59, with an average of 46±1. Clutch and egg volumes (cm^3^) ranged respectively from 223 to 306 (mean = 256.22±4.74) and from 74 to 102 (mean = 85.41±1.58). Female weight (g) ranged from 936 to 1100, with an average of 1005.17±11.28.

### 

#### Female fluctuating asymmetry, body condition and plasma anti-AIV antibody level

Descriptive statistics of signed (*l–r*) values for each of the four measured morphological traits are shown in Table 1. No trait had an average (*l–r*) that significantly departed from zero (*P*>0.05 for all comparisons, Table 1). Furthermore, for all traits the signed (*l–r*) values did not deviate significantly from a normal distribution (*P*>0.05 for all traits, Table 1). Overall, these results would suggest that in all traits the observed asymmetry was fluctuating and that no problem of directional asymmetry or antisymmetry occurred in the data. The composite fluctuating asymmetry index (FA), obtained by summing the calculated size-corrected fluctuating asymmetry values across the four traits, ranged from 0.016 to 0.087, with a mean value of 0.046±0.005.

**Table 1:** Statistical properties of signed differences between the right and left sides of female yellow-legged gulls for the four measured morphological traits and results of tests of normality and mean difference with zero. Sample size = 18.

Trait	Mean±SE	Skewness	Kurtosis	Student *t*-test for «mean = 0»		Shapiro-Wilk normality test	
				*t*	*P*	*W*	*P*
**Lower mandible length (mm)**	0.09±0.18	−0.29	−0.65	0.50	0.6218	0.97	0.7045
**Nalospi length (mm)**	0.13±0.07	−1.02	3.02	1.80	0.0899	0.91	0.0648
**Tarsus length (mm)**	−0.11±0.22	−0.34	1.10	−0.50	0.6243	0.95	0.4818
**Middle toe length (mm)**	−0.58±0.30	1.12	2.99	−1.96	0.0661	0.92	0.1135

In order to obtain a single estimate of female body condition, the Principal Component Analysis conducted on the three original variables (i.e., hematocrit value, clutch volume and size-corrected weight) allowed us to summarise these variables into one factor accounting for 62% of the original variance (Table 2). The remaining factors had eigenvalues less than 1, and were not retained (Table 2). The first factor derived from this PCA provided one composite index of body condition (BCI) that is positively correlated with female hematocrit value (*r* = 0.694, *P* = 0.0014), clutch volume (*r* = 0.772, *P*<0.0002) and size-corrected body weight (*r* = 0.889, *P*<0.0001). Females with the highest BCI values thus had the highest hematocrit values, the largest clutch sizes, and greatest size-corrected mass.

**Table 2:** Factors derived from the PCA of the three original condition descriptors.

Factor	Eigenvalue	Variance explained (%)	Cumulative variance (%)
1	1.87	0.62	0.62
2	0.77	0.26	0.88
3	0.36	0.12	100

**Figure 1:**
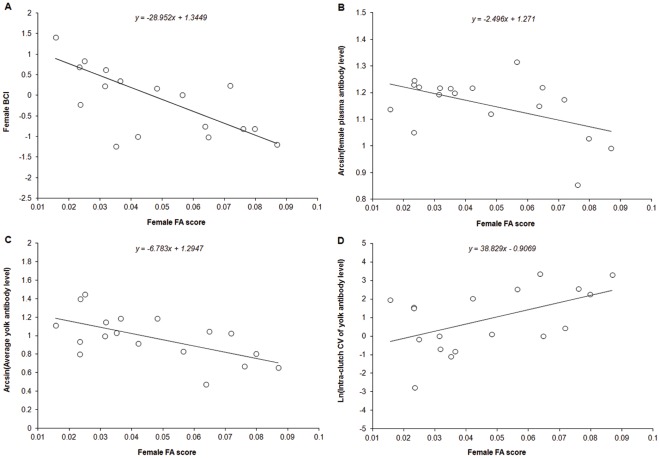
Relationship between female fluctuating asymmetry and (A) female body condition index, (B) anti-AIV antibody level in female plasma, (C) average yolk anti-AIV antibody level, and (D) intra-clutch CV of yolk anti-AIV antibody levels. The lines shown are the linear regression lines.

The results of the regression analyses show that female BCI was negatively associated with FA (*r^2^* = 0.43, *β*±SE = −28.95±8.34, *F*_1,16_ = 12.04, *P* = 0.0032, Fig.1-A). We also found that anti-AIV antibody levels in female plasma were negatively related to FA (*r^2^* = 0.26, *β*±SE = −2.50±1.05, *F*_1,16_ = 5.63, *P* = 0.0306, Fig.1-B), but were not correlated with BCI (*r^2^* = 0.08, *β*±SE = 0.03±0.03, *F*_1,16_ = 1.37, *P* = 0.2588). These results would suggest that females demonstrating greater asymmetry are also characterized by poorer body condition and lower plasma levels of anti-AIV antibodies than the more symmetric ones.

#### Egg antibody level in relation to female FA and laying order

**Figure 2:**
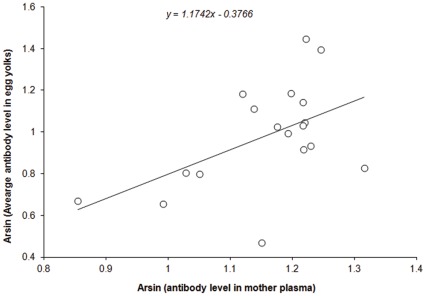
Mean egg antibody level as a function of plasma antibody level across all females sampled. The line shown is the linear regression line.

A positive relationship between the average level of anti-AIV antibodies in egg yolks and the level of anti-AIV antibodies in the plasma of the corresponding mother was found (*r^2^* = 0.26, *β*±SE = 1.17±0.49, *F_1,16_* = 5.80, *P* = 0.0285, Fig. 2). Clutches laid by females with higher levels of anti-AIV antibodies in their plasma received more antibodies than those laid by females with lower levels of anti-AIV antibodies. Furthermore, no significant relationship was found between female BCI and average yolk antibodies (*r^2^* = 0.13, *β*±SE = 0.09±0.06, *F_1,16_* = 2.42, *P* = 0.1390). We also found that the average level of anti-AIV antibodies in egg yolks was negatively related to maternal FA (*r^2^* = 0.36, *β*±SE = −379.77±124.68, *F_1,16_* = 9.28, *P* = 0.0077, Fig. 1-C), yet no significant correlation was found between female FA and the residuals of the regression of average egg antibody level on female antibody level (n = 18, *r* = −0.40, *P* = 0.10). Taken together, these results suggest that the eggs of more asymmetric females contain fewer antibodies because they are less competent at producing antibodies.

Within a given clutch, yolk anti-AIV antibody level varied significantly according to egg laying order (Table 3). The level of anti-AIV antibodies in the yolk decreased with the order in which an egg was laid (average values (%): 83±3 for egg 1, 80±4 for egg 2 and 78±4 for egg 3). However, only the difference between the first and the third egg was significant (post-hoc Duncan, *P*<0.05). These results would suggest that, within a given clutch, the amount of antibodies received from the mother decreased gradually from the first-laid to the last-laid egg.

**Table 3:** Results of repeated-measure ANOVA on the yolk level of anti-AIV antibodies as a function of egg laying order. Model R^2^ = 0.89, F_19,34_ = 14.87, *P*<0.0001.

Effect	DF	Type III SS	F	P
Nest identity	17	10268.155	16.23	<0.0001
Laying order	2	242.267	3.26	0.0509

Our results also reveal an effect of female FA on the laying order pattern of antibody transfer. The results of a mixed model in which yolk antibody was a function of FA and laying order, with female identity as a random effect, showed a significant interaction between female FA and egg laying order (*β* = −0.98±0.44, *F_2,32_* = 5.13, *P* = 0.0316). The nature of this interaction was revealed by further tests. In investigating the relationships between female FA and yolk antibody level for first, second and third eggs, we found that yolk antibodies decreased with increasing female FA regardless of the laying order of sampled eggs (Egg 1: *r^2^* = 0.36, *β*±SE = −6.26±2.08, *F_1,16_* = 9.04, *P* = 0.0084; Egg 2: *r^2^* = 0.30, *β*±SE = −6.46±2.46, *F_1,16_* = 6.88, *P* = 0.0185; Egg 3: *r^2^* = 0.38, *β*±SE = −7.74±2.46, *F_1,16_* = 9.89, *P* = 0.0063). This means that more asymmetric females transfer fewer antibodies to their eggs from the beginning. Furthermore, we found that the extent of within-clutch variation in yolk anti-AIV antibodies (intra-clutch CV) was positively correlated with mother FA (*r^2^* = 0.26, *β*±SE = 38.83±15.98, *F_1,16_* = 2.43, *P* = 0.027, Fig. 1-D), but the positive relationship between the difference in antibody level between the first and the last egg and FA was marginally significant (*r^2^* = 0.17, *β*±SE = 10.39±5.72, *F_1,16_* = 3.30, *P* = 0.0881). These latter results would suggest that the laying order effect was more pronounced in the clutches laid by the more asymmetric females.

## Discussion

In this study, we used the yellow-legged gull study system to investigate how female quality, as estimated by fluctuating asymmetry, interacts with egg laying order to shape patterns of antibody transmission. No attention has previously been paid to the link between a female’s morphological asymmetry and her capacity to produce and transmit antibodies into the eggs. Overall, our results support the hypothesis that clutches laid by asymmetric females received fewer antibodies and exhibited a more pronounced laying order effect than those laid by the more symmetric ones. However, because our results are based on a small sample size (n = 18 positive females), the results we found should be interpreted with caution. More data are needed for firmer conclusions about the association between patterns of maternal antibody transfer and mother FA.

Both female body condition and plasma antibody level were found to be negatively correlated with the degree of fluctuating asymmetry. As fluctuating asymmetry is generally associated with a reduced ability to satisfy nutritional needs and face environmental challenges [18], [25], [45], [46], the negative relationship between fluctuating asymmetry and body condition was expected. Furthermore, because individuals facing stressful conditions may be unable to mount an efficient immune response [47], the negative relationship between female fluctuating asymmetry and plasma antibody level was also expected. The latter result stresses once more that higher amounts of plasma immunoglobulins often characterize females of higher phenotypic quality [6]. Surprisingly, female body condition was not correlated with antibody levels in either female plasma and egg yolks.This result seems to be in contradiction with the findings of previous studies showing that humoral immucompetence is strongly related to female body condition during egg production [48]. This result may signify that fluctuating asymmetry provides a better predictor of female quality and thus plasma antibody levels because it takes into account the longer term developmental history of the females studied; in contrast, the simple measure of body condition may instead reflect a shorter term snapshot of current breeding conditions. Diminished body condition and limited humoral immunocompetence should both be viewed as consequences of developmental instability due to stressful conditions during early life stages.

Our results show that the level of antibodies in the eggs was positively correlated with the level of antibodies in their mother’s plasma. This finding is consistent with the general trend that females with higher levels of antibodies in their plasma transmit more antibodies to their eggs [7], [49], whether passively or actively. Moreover, our results show that eggs laid by asymmetric females contained fewer antibodies than those laid by the more symmetric ones. Under the hypothesis that antibody transfer is a passive mechanism, such a pattern could be a direct consequence of differences among females in their capacity to produce antibodies. Alternatively, it could result from differences in antibody transmission capacity, i.e. asymmetric females transmitted fewer antibodies than predicted by the levels in their plasma. Our results support the former rather than the latter argument: when female antibody level was controlled for, the correlation between egg antibody level and female fluctuating asymmetry disappeared. Our results thus suggest that eggs laid by asymmetric females contained fewer antibodies than those laid by more symmetric females because of maternal differences in antibody production.

These findings highlight the fact that stressful developmental conditions that females suffer during their early life stages can not only negatively affect their phenotype, but also those of their offspring. Asymmetric females may face a greater selective disadvantage compared to more symmetric ones because, in addition to their own diminished body condition and immunocompetence, their offspring also receive lower immune protection and are, consequently, exposed to higher mortality risks. Indeed, maternal antibodies are known to provide the young with a protection against pathogens during critical life stages [50], [51]. Low passive immune protection is generally associated with high chick mortality in birds [9], [52], [53].

Our results also show a strong laying order effect for yolk antibodies within clutches. The amount of antibodies in the yolk gradually decreased with egg laying order. This result is consistent with previous findings from related gull species [10], [11]. It also supports the hypothesis that the yellow-legged gull adopts the brood reduction strategy [29]. By allocating less immunity to the last-laid egg, females may be enhancing the survival of earlier hatched, more reproductively valuable nestlings while lowering the survival prospects of the nestling with the lowest reproductive value (the last nestling) [15], [16]. More interestingly, we found that the degree to which yolk antibodies decrease with laying order within the clutch increases with maternal fluctuating asymmetry. Differences in antibody transmission seem to be more pronounced in the more asymmetric females compared to the symmetric ones. However, our results also suggest that more asymmetric females transfer fewer antibodies to their eggs from the beginning, and that the decline in yolk antibodies with increasing female asymmetry could not entirely be attributed to laying order effects. Last eggs laid by the most asymmetric females received fewer antibodies not only because of their last laying rank but also because they were laid by low-quality females. Overall, these results support the hypothesis that the brood reduction strategy seems to be more acutely manifest in clutches laid by the more asymmetric females. Further investigations of how egg laying order and female fluctuating asymmetry relate to chick immunocompetence, growth, and survival in other species and other reproductive systems would tell us more about this issue. In particular, an experimental approach that includes a controlled exposure of females to an antigen would help determine if the reported relationships show cause and effect.

It should be noted that our findings may have been partly biased by differences in infection history among the sampled females. Because nothing was known about the infection history of the studied females, it is impossible to know whether females with high levels of antibodies were good responders, i.e. able to mount a strong, durable immune response to infection, or recently infected relative to females with low levels of antibodies. While we recognize that these unknowns may have introduced some bias in our results, we believe that they are unlikely to drive the observed trend: more asymmetric females had lower levels of anti-AIV antibodies than the more symmetric ones. It is improbable that females with high levels of FA (and low levels of antibodies) were all old responders while females with low FA levels (and high levels of antibodies) were all recent responders. Nonetheless, we believe that future studies should utilize experimental approaches. For instance, vaccinating females with different levels of FA would more accurately reveal the relationship between female quality and antibody production and transmission. One further factor that may be hypothesized to affect female antibody level and patterns of maternal antibody transfer is female age. Our study sample was entirely composed of early-nesting females that could be considered to be among the oldest females in the study colony [54]. Our interpretation is thus restricted to this age set and a larger dataset incorporating a wider age range would be necessary to investigate the broader applicability of our results.

In conclusion, the results of our work show the great complexity of factors affecting the transmission of maternal antibodies to eggs in birds. In particular, they stress the need to consider female developmental history when dealing with the ecology and evolution of immunity transfer. They also highlight the usefulness of fluctuating asymmetry as a measure of individual quality and immunocompetence.

## References

[1] Dobson A, Lafferty KD, Kuris AM, Hechinger RF, Jetz W (2008). Homage to Linnaeus: How many parasites? How many hosts?. Proc Natl Acad Sci USA.

[2] Grindstaff JL, Brodie ED, Ketterson ED (2003). Immune function across generations: integrating mechanism and evolutionary process in maternal antibody transmission.. P Roy Soc Lond B Bio.

[3] Boulinier T, Staszewski V (2008). Maternal transfer of antibodies: raising immuno-ecology issues.. Trends Ecol Evol.

[4] Hasselquist D, Nilsson JÅ (2009). Maternal transfer of antibodies in vertebrates: Trans-generational effects on offspring immunity.. Phil Trans R Soc B.

[5] Klasing KC (1998). Nutritional modulation of resistance to infectious diseases.. Poult Sci.

[6] Saino N, Martinelli R, Møller AP (2001). Immunoglobulin plasma concentration in relation to egg laying and mate ornamentation of female barn swallows (*Hirundo rustica*).. J Evol Biol.

[7] Grindstaff JL, Demas GE, Ketterson ED (2005). Diet quality affects egg size and number but does not reduce maternal antibody transmission in Japanese quail *Coturnix japonica*.. J Anim Ecol.

[8] Hargitai R, Prechl J, Török J (2006). Maternal immunoglobulin concentration in collared flycatcher (*Ficedula albicollis*) eggs in relation to parental quality and laying order.. Funct Ecol.

[9] Buechler K, Fitze PS, Gottstein B, Jacot A, Richner H (2002). Parasite-induced maternal response in a natural bird population.. J Anim Ecol.

[10] Blount JD, Surai PF, Nager RG, Houston DC, Møller AP (2002). Carotenoids and egg quality in the lesser black-backed gull *Larus fuscus*: a supplemental feeding study of maternal effects.. Proc R Soc B.

[11] Müller W, Groothuis TGG, Dijkstra C, Siitarni H, Alatalo RV (2004). Maternal antibody transmission and breeding densities in the Black-Headed Gull *Larus ridibundus*.. Funct Ecol.

[12] Slagsvold T, Sandvik J, Rofstad G, Lorentsen O, Husby M (1984). On the adaptive value of intraclutch egg-size variation in birds.. Auk.

[13] Schwabl H (1993). Yolk is a source of maternal testosterone for developing birds.. Proc Natl Acad Sci USA.

[14] Lipar JL, Ketterson ED, Nolan V (1999). Intra-clutch variation in testosterone content of red-winged blackbird eggs.. Auk.

[15] Schwabl H, Mock DW, Gieg JA (1997). A hormonal mechanism for parental favouritism.. Nature.

[16] Gil D, Graves J, Hazon N, Wells A (1999). Male attractiveness and differential testosterone investment in zebra finch eggs.. Science.

[17] Watson PJ, Thornhill R (1994). Fluctuating asymmetry and sexual selection.. Trends Ecol Evol.

[18] Møller AP, Swaddle JP (1997). Developmental Stability & Evolution..

[19] Parsons PA (1990). Fluctuating asymmetry: an epigenic measure of stress.. Biol Rev.

[20] Fair JM, Hanssen ES, Ricklefs RE (1999). Growth, developmental stability and immune response in juvenile Japanese quail (*Coturnix coturnix japonica*).. Proc R Soc Lond B.

[21] Brown CR, Brown MB (2002). Ectoparasites cause increased bilateral asymmetry of naturally selected traits in a colonial bird.. J Evol Biol.

[22] Møller AP (2006). A review of developmental instability, parasitism and disease.. Infect Genet Evol.

[23] Amat JA, Aguilera E, Visser GH (2007). Energetic and developmental costs of mounting an immune response in greenfinches (*Carduelis chloris*).. Ecol Res.

[24] Møller AP (1999). Condition-dependent asymmetry is fluctuating asymmetry.. J Evol.

[25] Polak M (2003). Developmental Instability: Causes and Consequences..

[26] Mateos C, Alarcos S, Carranza J, Sánchez-Prieto CB, Valencia, J (2008). Fluctuating asymmetry of red deer antlers negatively relates to individual condition and proximity to prime age.. Anim Behav.

[27] Cramp S (1998). The Birds of the Western Palearctic on CD Rom..

[28] Rubolini D, Romano M, Martinelli R, Saino N (2006). Effects of elevated yolk testosterone levels on survival, growth and immunity of male and female yellow-legged gull chicks.. Behav Ecol Sociobiol.

[29] Saino N, Bertacche V, Bonisoli-Alquati A, Romano M, Rubolini D (2008). Phenotypic Correlates of Yolk and Plasma Carotenoid Concentration in Yellow-Legged Gull Chicks.. Physiol Biochem Zool.

[30] Bosch M, Oro D, Cantos FJ, Zabala M (2000). Short-term effects of culling on the ecology and population dynamics of the Yellow-legged Gull.. J Appl Ecol.

[31] Vidal E, Médail F, Tatoni T (1998). Is the Yellow-legged Gull a superabundant species in the Mediterranean? Impact on fauna and flora, conservation measures and research priorities.. Biodivers Conserv.

[32] Oro D, Martinez-Abrain A (2007). Deconstructing myths on large gulls and their impact on threatened sympatric waterbirds.. Anim Conserv.

[33] Cramp S, Simmons KEL (1983). Handbook of the Birds of Europe, the Middle East and North Africa: The Birds of the Western Palearctic, Volume III..

[34] Isenmann P, Gaultier T, Hili A, Azafzaf H, Dlensi H (2005). Oiseaux de Tunisie..

[35] Potti J, Moreno J, Merino S, Frias O, Rodriguez R (1999). Environmental and genetic variation in the haematocrit of fledgling pied flycatchers *Ficedula hypoleuca*.. Oecologia.

[36] Hoi-Leitner M, Romero-Pujante M, Hoi H, Pavlova A (2001). Food availability and immune capacity in serin (*Serinus serinus*) nestlings.. Behav Ecol Sociobiol.

[37] Griffiths R, Double MC, Orr K, Dawson RJG (1998). A DNA test to sex most birds.. Mol Ecol.

[38] Fridolfsson AK, Ellegren H (1999). A simple and universal method for molecular sexing of non-ratite birds.. J Avian Biol.

[39] Mohammed HO, Yamamoto R, Carpenter TE, Ortmayer HB (1986). Comparison of egg yolk and serum for the detection of *Mycoplasma gallisepticum* and *M. synoviae* antibodies by enzyme-linked immunosorbent assays.. Avian Dis.

[40] Gasparini J, McCoy KD, Haussy C, Tveraa T, Boulinier T (2001). Induced maternal response to the Lyme disease spirochaete *Borrelia burgdorferi sensu lato* in a colonial seabird, the kittiwake *Rissa tridactyla*.. P Roy Soc Lond B.

[41] Palmer AR (1994). Fluctuating asymmetry analyses: A primer.. Markow TA, ed.

[42] Harris MP (1964). Aspects of the breeding biology of the gulls *Larus argentatus, L. fuscus and L. marinus.*. Ibis.

[43] Bolton M, Monaghan P, Houston DC (1993). Proximate determination of clutch size in lesser black-backed gulls: the roles of food supply and body condition.. Can J Zool.

[44] SAS Statistical Institute (1998). SAS/STAT user’s guide, version 8..

[45] Balmford A, Jones IL, Thomas ALR (1993). On avian asymmetry: evidence of natural selection for symmetrical tails and wings in birds. Proc R Soc Lond B..

[46] Thomas ALR (1993). The aerodynamic costs of asymmetry in the wings and tails of birds: asymmetric birds can’t fly round tight corners.. Proc R Soc Lond B.

[47] Read AF, Allen JA (2000). The economics of immunity.. Science.

[48] Pihlaja M, Siitari H, Alatalo RV (2006). Maternal antibodies in a wild altricial bird: effects on offspring immunity, growth and survival.. J Anim Ecol.

[49] Gasparini J, McCoy KD, Tveraa T, Boulinier T (2002). Related concentrations of specific immunoglobulins against the Lyme disease agent *Borrelia burgdorferi sensu lato* in eggs, young and adults of the kittiwake (*Rissa tridactyla*).. Ecol Lett.

[50] Klasing KC, Leshchinsky TV (1999). Function, costs and benefits of the immune system during development and growth.. Adams NJ & Slotow RH, eds.

[51] Grindstaff JL (2008). Maternal antibodies reduce costs of an immune response during development.. J Exp Biol.

[52] Heeb P, Werner I, Kölliker M, Richner H (1998). Benefits of induced host responses against an ectoparasite.. Proc R Soc B.

[53] Sahin O, Luo ND, Huang SX, Zhang QJ (2003). Effect of Campylobacter-specific maternal antibodies on *Campylobacter jejuni* colonization in young chickens.. Appl Environ Microbiol.

[54] Sydeman WJ, Penniman JF, Penniman TM, Pyle P, Ainley DG (1991). Breeding performance in the Western gull–Effects of parental age, timing of breeding and year in relation to food availability.. J Anim Ecol.

